# A Community–School District–University Partnership for Assessing Physical Activity of Tweens

**Published:** 2008-12-15

**Authors:** Robert J. McDermott, Jen Nickelson, Julie A. Baldwin, Carol A. Bryant, Moya Alfonso, Leah M. Phillips, Rita D. DeBate

**Affiliations:** Florida Prevention Research Center, University of South Florida, College of Public Health; University of South Florida, Tampa, Florida; University of South Florida, Tampa, Florida; University of South Florida, Tampa, Florida; University of South Florida, Tampa, Florida; University of South Florida, Tampa, Florida; University of South Florida, Tampa, Florida

## Abstract

**Introduction:**

Obesity among youth is related to a decline in physical activity, and data on physical activity levels among children in elementary and middle schools are limited.

**Methods:**

We leveraged a community–school district–university partnership in Sarasota County, Florida, in May of 2005 to assess physical activity levels among tweens (youth aged 9-13 years) and to measure the relationship between tweens' awareness of the Centers for Disease Control and Prevention's VERB program and participation in physical activity, using a minimally obtrusive survey. After surveying participating schools (4 elementary schools and 3 middle schools), we obtained 1,407 responses from children in grades 5 through 7.

**Results:**

In all, 83.1% of students met the federal recommendation for daily participation in vigorous-intensity physical activity (VPA), and 58.6% had tried a new game or sport within the previous 2 months. Mean number of days in the previous week engaging in VPA was significantly higher (*P* < .001) for boys (5.22) than for girls (4.35). Mean number of days engaging in VPA in the previous week was significantly higher (*P* = .006) among 6th-grade students (4.93) than 7th-grade students (4.54), but no consistent decline through the grade levels occurred. Activity was significantly correlated with the number of friends reported as playing a game or sport daily (*r* = .369, *P* < .001). Most students (88.8%) reported having seen, read, or heard messages or ads about VERB, a tween-centric national social marketing campaign promoting physical activity and participation in new games and sports.

**Conclusion:**

Although participation in VPA was high, girls reported significantly fewer days spent engaged in VPA than did boys. We found a modest association between engaging in VPA and having active friends. Capitalizing on leadership from multiple community-based organizations to monitor youth physical activity may inspire implementation of strategies for motivating youth to try new games and sports that they can sustain through the adolescent years and beyond.

## Introduction

The benefits of physical activity for adolescents are many (1). Physiological benefits include less body fat, reduced blood pressure in mildly hypertensive youth, and improvements in cardiovascular fitness and skeletal health. Physical activity improves mental health and academic performance (1,2). Conversely, physical inactivity is associated with increased all-cause mortality (3) and increased body fat (4). Childhood obesity is a public health concern because it may be a precursor to adult obesity (5,6).

Behaviors that contribute to obesity, such as physical inactivity, are established during childhood (6). Therefore, increasing the proportion of children and youth who meet physical activity guidelines is important for primary prevention (7). National recommendations for participation in physical activity indicate that adolescents should engage in at least 30 minutes of moderate-intensity physical activity on 5 or more days per week or in at least 20 minutes of vigorous-intensity physical activity (VPA) on 3 or more days per week (8). More recently, a new recommendation has emerged advising 60 or more minutes of daily, moderate- to vigorous-intensity physical activity for youth (1).

The Centers for Disease Control and Prevention's (CDC's) Youth Risk Behavior Surveillance System (YRBSS) is conducted biennially among adolescents to assess the prevalence of health-promoting and risk behaviors, including physical inactivity; however, these data are collected only among high school students (9). Less information is available about physical activity behaviors of children in elementary and middle schools.

Development of tools that assess children's physical activity levels has been identified by some authorities as a research priority (10). Self-report methods often are used because they are inexpensive and easy to administer (11). However, if children are asked to recall activity that occurred too far from the time of data collection (10), accuracy of measurement can be compromised, usually resulting in overestimation (12,13), a response bias possibly due to social desirability (14). Overreporting may also be due in part to seasonally related issues (15). This concern notwithstanding, multiple research studies support self-report's accuracy with respect to physical activity for youth, particularly in grades 7 and higher (16,17). A challenge for school health personnel is to monitor physical activity in ways that are neither obtrusive for the children involved nor taxing or too complex for teachers, school nurses, or others who are asked to make these assessments.

Finding effective ways to address the decline in physical activity among youth has become a priority of CDC. CDC's VERB program was a national media campaign that ran from 2002 to 2006 to increase physical activity among tweens (children aged 9-13 years) and help them maintain physical activity levels (18). VERB was promoted in diverse ways (eg, paid media advertising, public service announcements, special activity promotions) (18). Within schools, VERB was promoted through the *Weekly Reader*, *TIME for Kids*, *ChannelOne*, and other materials (18). Marketing firms assisted in developing and promoting this edgy, tween-centric media campaign — the purpose of which is to popularize physical activity with tweens by making it cool and fun, an approach not unlike ones used to market other popular tween commodities.

In Sarasota County, Florida, the Sarasota County Obesity Prevention Coalition was established in May 2003 as an advocacy and needs-assessment group for promoting health behaviors and disseminating information about the impact of obesity on quality of life. Represented on the coalition are the county school board, the county health department, the county parks and recreation department, the YMCA (Young Men's Christian Association), and various youth-centered and health-focused organizations. The coalition invited the Florida Prevention Research Center at the University of South Florida to help guide some of its initiatives that have youth as a priority population. Stakeholders chose physical activity as the health behavior of interest because of its documented relationship to overweight among youth and because stakeholders recognized that leisure-time interests that are popular among children (eg, watching television, playing video games) promote being sedentary. Furthermore, the coalition recognized the opportunity to capitalize on the popularity of the VERB media campaign by adapting local programming to the aims of the campaign. The research environment provided by participating schools, combined with the research expertise of the university faculty, provided an ideal setting in which to generate data for the community.

The purpose of this study was to assess physical activity among tweens and to measure the relationship between tweens' awareness of CDC's VERB program and participation in physical activity.

## Methods

### Setting and overview

Sarasota County, Florida, is located on the southwest coast of the state and has a population of approximately 360,000 (19). Although the county is characterized as a prime location for retirees, the young population is growing. Youth younger than 18 years comprised 16.9% of the county population in 2005, compared with 16.2% in 2000 (19). Most residents (90.7%) are white, and a small percentage (4.0%) are black (23). The median household income in the county ($44,505) is between that of Florida overall ($42,433) and the United States as a whole ($46,242) (19).

### Participants

In May of 2005, approximately 9,500 public school students were in grades 5 through 7 in Sarasota County, Florida. We conducted a power analysis, which indicated that the minimum sample size for detecting differences in physical activity level by sex and grade with α = .05, β = .20, and power = .80 was N = 782. We sought to obtain a sample size twice as large as needed to account for absenteeism and refusals. We randomly selected schools from the pool of middle schools containing only grades 6 through 8 (N = 5) and elementary schools containing grades kindergarten through 5 (N = 20). Ultimately, participants came from 3 middle schools and 4 elementary schools. These schools varied in size and geographic location. The total estimated census for the grades selected from these schools was 1,590. All students in grades 5, 6, and 7 present in these schools at the time of survey administration were included without duplication. The setting was the classroom.

### Survey instrument

Taking the age of respondents and the burden on participating schools into consideration, a panel of experts in physical activity, school health, educational measurement, and child development produced a brief self-report survey instrument for the priority age group. In addition to demographic items of sex and grade, constructs included number of days per week engaged in VPA, perceived peer engagement in physical activity, frequency of trying a new physical activity, and awareness of VERB and a derivative program, VERB Summer Scorecard. The survey contained 1 additional item about another of the county's youth-focused programs, unrelated to physical activity.

The item concerning participation in VPA was worded similarly to one used in the YRBSS (20): "On how many of the last 7 days did you play a game or sport (like running, basketball, soccer, swimming, biking, or other fun thing) for 20 minutes or more that caused you to sweat or to breathe hard?" Students could select from 8 possible responses (0 days to 7 days).

Three items from the Youth Media Campaign Longitudinal Survey (21) were revised slightly for inclusion in the survey. The peer-social normativeness of physical activity and sports was measured by the question, "How many of your friends play a game or a sport (like running, basketball, soccer, swimming, biking, or other fun thing) every day?" Possible responses were "none or just a few," "some," or "most or all." The frequency of trying a new activity was measured by the question, "In the last 2 months, have you tried a new game or sport (rock climbing, roller blading, or other fun thing) that you've never done before?" Possible responses were yes or no. Awareness of the VERB and VERB Summer Scorecard programs was measured by the questions, "Have you ever seen, read, or heard any messages or ads about VERB?" and "Have you ever seen, read, or heard any messages or ads about VERB Summer Scorecard?" Possible responses to these to items were yes or no.

Items were pilot tested one-on-one with 7 children aged 9 to 13 years to assess face validity and comprehension (22), both of which were concluded to be satisfactory. The final product, reviewed and approved by the coalition, was a 1-page, 8-item survey that could be completed in 5 minutes or less. The survey yielded a Gunning-Fog Index of 5.91 (23), a Flesch-Kincaid Grade Level score of 4.16 (24), and a Flesch Reading Ease score of 86.10 (24), all of which suggest that the survey was easy to read at approximately the 5th-grade level. Test-retest reliability of the survey (n = 15) over a 14-day period produced item percentage agreements ranging from 67% (days of physical activity over the past week) to 100% (demographic items, awareness of VERB programs).

### Survey administration

We prepared packets of 25 surveys to distribute to school nurses, who were briefed on the protocol for administering the surveys. Each packet was color-coded by school and contained a set of directions for people facilitating the process to read aloud to students, assuring them of the anonymity and confidentiality of their responses and informing them of their right to withdraw from participating in the study at any time without fear of any negative consequences. Both teachers and students are accustomed to school nurses' presence in the classroom, making them appropriate partners for survey research; nurses and teachers administered the surveys in classrooms. The participating school district uses a passive parental permission protocol that was established at the beginning of the school year for surveys of this type. Consequently, parents would have to make a specific request that their child be excluded from participating in the survey. Thus, virtually all students present at school when surveys were administered participated in data collection. All students consented to participate. They were instructed to return the survey to an unmarked envelope upon completion. The envelope was sealed until data entry. The instrument and its administration protocol received review and approval by the institutional review board of the University of South Florida.

### Data analysis

Data were entered into a spreadsheet and analyzed using SPSS version 13.0 for Windows (SPSS Inc, Chicago, Illinois). Univariate analyses were conducted to provide descriptive and demographic data. Bivariate statistical procedures were Spearman rank correlation, 1-way analysis of variance (ANOVA), and *t *test. A Tukey post-hoc test was conducted to identify sources of difference found by the ANOVA procedure. Statistical significance was set at .05, and 95% confidence intervals (CIs) were calculated.

## Results

### Demographics

The estimated census of students for the participating schools was 1,590. In all, 1,443 surveys were returned. All students consented to participate in the survey, but 36 responses were excluded from the analysis because of missing data. Thus, 720 girls (51.2%) and 687 boys (48.8%) comprised the group of 1,407 students who completed the survey. Of students in our sample, 315 (22.4%) were in the 5th grade, 578 (41.1%) were in the 6th grade, and 514 (36.5%) were in the 7th grade (Table 1). Data in Table 2 show the number days per week children reported engaging in VPA.

Students whose surveys were excluded from the analysis because of the amount of missing data were not significantly different from the included students with respect to sex, grade, and current reported physical activity level. One elementary school had a significantly higher proportion of students with missing data (8.6%) than other schools (data not shown).

### Physical activity level

Overall, students engaged in VPA an average of 4.77 days per week (Table 3). Mean number of days per week engaged in VPA was significantly higher among boys than among girls (5.22 days vs 4.35 days, *t* = 7.97, *P <* .001) (Table 1). The results of a 1-way ANOVA revealed a significant difference by grade (F = 5.12, *P =* .006) (data not shown). Tukey post-hoc analysis revealed the mean number of days engaged in VPA among 6th-grade students was significantly higher than the mean for 7th-grade students (4.93 days vs 4.54 days, *P* = .006) (Table 1). Approximately one-third of children reported engaging in VPA every day of the week (n = 445, 31.6%). Approximately 59.2% of the students reported participating in VPA on 5 or more days of the previous 7 days, and 83.1% reported VPA on 3 or more days per week.

### Perceived social norms

Most students (n = 648, 46.1%) responded that "most or all" of their friends play a game or a sport every day of the week (Table 3). The number of friends students reported as playing a game or sport every day was significantly correlated with the number of days per week students were engaged in VPA (*r* = .369, *P* < .001). Students who reported that "most or all" of their friends played a game or sport every day engaged in VPA an average of 5.53 days per week. Students reporting that "some" of their friends played a game or sport every day engaged in VPA an average of 4.31 days per week, and students who reported that "none or just a few" of their friends played a game or sport every day engaged in VPA an average of 3.39 days per week.

A total of 825 (58.6%) students reported they had tried a new game or sport in the last 2 months, and 582 (41.4%) reported they had not. Students who reported having tried a new game or sport in the last 2 months engaged in VPA a significantly higher mean number of days per week than students who reported they had not tried a new game or sport (5.14 days vs 4.25 days, *t* = 7.81, *P* < .001).

### VERB and VERB Summer Scorecard messages

Most students surveyed (n = 1,250, 88.8%) reported that they had seen, read, or heard messages or ads about VERB. Students who reported VERB awareness engaged in VPA a significantly higher mean number of days per week than students who were unfamiliar with VERB (4.82 days vs 4.37 days; *t* = −2.56, *P =* .01). However, there was very slight overlap in the 95% CIs for students familiar with (95% CI, 4.70-4.94) and not familiar with (95% CI, 4.03-4.71) VERB (Table 3).

Most students (n = 1022, 72.6%) reported not having been exposed to VERB Summer Scorecard messages. Students who reported familiarity with VERB Summer Scorecard engaged in VPA a significantly higher mean number of days per week than students who were unfamiliar with this program (4.99 days vs 4.69 days; *t* = −2.39, *P =* .02) (Table 3). Once again, there was overlap in the 95% CIs.

## Discussion

The percentage of children in this study who participated in VPA on 3 or more days per week (83.1%) approximates the goal of 85% identified in *Healthy People 2010* (8) for adolescents in grades 9 through 12. However, our study participants were in grades 5 through 7. Sallis (25) points out that participation in physical activity declines among youth over time. Our data showed variation in mean days engaged in VPA in grades 5 through 7 but did not support a downward trend (ie, 6th-grade students reported more days engaged in VPA days than 7th-grade students, but CIs for 5th- and 7th-grade students overlapped, as did those for 5th- and 6th-grade students). Although data about physical activity levels of younger children are generally lacking, those that are available suggest that age-related declines may begin between the ages of 12 and 15 years (25). Previous research estimated the proportion of Florida high school youth who met the criteria for participation in VPA to be 60.8% (26). Thus, having innovative mechanisms for maintaining youth interest in physical activity through all grade levels is critical for the fight against childhood obesity to be successful. Interventions such as VERB that make physical activity "fun and cool" may be part of a genre of initiatives for motivating youth to be active, and this is supported by research involving the PARADE study (27). Local adaptations of VERB or other endeavors may help retard the current decline in physical activity during adolescence. Unfortunately, VERB has not been in CDC's Congressionally sponsored budget since the end of September 2006 (28,29), so its ability to have any future residual influence on youth behavior remains to be seen.

*Healthy People 2010* (8) objectives have identified increasing the proportion of adolescents participating in daily school physical education as an appropriate behavior change to offset the rise in childhood obesity and combat decline in physical activity over time. Regular physical education attendance can influence moderate-intensity and vigorous-intensity levels of physical activity, as well as affect knowledge, attitudes, and skills for lifelong physical activity (30). Initiatives to restore and increase physical education requirements in grades kindergarten through 12 are worthwhile endeavors for school health advocates to augment and bolster other community-based programs.

Our research demonstrated that boys reported engaging in VPA on more days than girls. Other studies have demonstrated this phenomenon as well (25,31-34). This finding is concerning because physical activity is a component of healthy bone development, including optimal bone density (35). The amount of mineralized bone established during adolescence is approximately equivalent to the amount lost throughout the remainder of adult life (36). Bone loss in women is a notable health problem, and the failure to achieve optimal bone mass during adolescence may compromise bone health later in life (35).

The finding that more than half of the respondents (58.6%) indicated they had tried a new game or sport in the past 2 months may be indicative of a positive influence of exposure to the VERB program. However, youth who already are active may be more attuned and responsive to physical activity promotion programs. Overall, students who were aware of VERB and VERB Summer Scorecard tended to be more active than students who were unaware of these programs, although the overlap in CIs for both groups indicate that these relationships may be tenuous. Although this study shows only that awareness and activity levels are associated, and one cannot be said to cause the other, already active tweens may be a priority audience segment on which to focus for sustaining physical activity through adolescence and into adulthood. Interestingly, a larger proportion of youth (89%) reported VERB campaign awareness in this community than the 74% that CDC researchers determined in a nationally based study (37). Most students reported not having been exposed to VERB Summer Scorecard messages, a finding not surprising given that survey administration occurred before the launch of the VERB Summer Scorecard program. Whereas some students may have been exposed to preannouncements about the program, others reporting message recognition may not have been distinguishing VERB from VERB Summer Scorecard.

The theory of planned behavior suggests that behaviors are influenced primarily by intentions and that intentions are influenced, in part, by perceived social norms (38,39). In our study, more active students reported that their friends also were active, participating in games or sports on a daily or near daily basis. One of the challenges of promoting physical activity is to make the benefits of participating in physical activity more attractive than the associated costs. CDC's extensive research with tweens revealed that tweens value "spending time with friends, playing, having fun, having an opportunity to be active with parents . . . gaining recognition from peers and adults . . . [and] the opportunity to explore and discover the world around them" (18).

This study has notable limitations. First, the sample was restricted to students in grades 5 through 7 who attend public schools in 1 county in Florida. This restriction may have contributed to not finding a downward trend in daily VPA through the grade levels. Perhaps such a result would have been noted if the cross-sectional sample had been extended to high school settings. Second, the brief survey did not contain a range of items that may have offered a more complete picture of physical activity and its determinants among children and youth. Third, the frequency of VPA reported may have depended on individual school physical education policy and whether survey participants were currently enrolled in physical education classes. Fourth, the data were produced through self-report, a process that can be influenced by the ability for accurate recall. Fifth, physical activity was assessed using a single item pertaining only to frequency; thus, no information was obtained about intensity or duration of actual activity. Consequently, construct validity cannot be assured. Sixth, test-retest reliability of an instrument can be influenced by number of participants, respondent bias, and time interval between administrations (40); therefore, the reported reliability of the survey used in this study may lack precision. Additionally, we did not assess moderate-intensity physical activity, which is most likely the level at which a large proportion of youth engage. Finally, we are aware of the revised physical activity recommendations for adolescents (1). However, this study was conducted before the revision, and it focused exclusively on VPA. Consequently, generalizing these findings to similar age groups and settings is imprudent.

The study was not without notable strengths. The approach had practical value because it offered school and community officials insight about at least 1 factor known to contribute significantly to youth obesity and to many chronic health problems in adulthood. To date, relatively few studies have examined physical activity in this age group. Schools of different sizes and from all parts of the geographic area participated. Moreover, the survey was at an appropriate reading level and used previously tested items. The protocol was carried out by teachers and school nurses, people whom students are accustomed to seeing regularly and with whom they are familiar; therefore, the experience was not intimidating to the students. In addition, the survey was short and easy to administer, so the burden on school personnel and students was minimal. Lessening the burden may have contributed to a high rate of participation by youth and to greater cooperation by school authorities. The survey's brevity probably also eliminated the type of respondent fatigue and resulting bias that is common with long and multithematic surveys that are resisted by students and school personnel. Another recent survey of school-aged youth required more labor-intensive efforts to obtain essential physical activity measures and needs-assessment data (41). Finally, the cooperation of a coalition and a school district, assisted by a university, demonstrated local interest in, and ownership of, a national health issue.

The university leveraged some of its financial resources and talents to support actions for combating a health problem already identified by the community. The formation of a community–school district–university partnership fostered recognition of at least 3 process-product relationships:

- The pooled time, talents, and other assets held by coalition partners exceed ones indigenous to any one of the partners.
- Careful stewardship of system resources enables an expanded base of services for the community.
- Resistance to cooperation virtually ceases when all stakeholder groups contribute to prioritizing outcomes.

In community–school district–university partnerships, process and product are critical elements. Focusing on developing mutually important outcomes early in the partnership is essential for nurturing empowerment, ownership, capacity, and trust. Creating visible products through a cooperative partnership (ie, identifying a health problem, creating a protocol to study it, developing and administering a survey, and preparing a report through which an evidence-based solution can be crafted) are necessary to build interest in other projects that can increase a community's capacity for solving its health problems. In our study, each partner was able to claim credit for the successful initiative, and in turn increase its own capacity, along with that of the entire coalition. Such a successful relationship among partners galvanized trust, respect, and commitment for the future. Using a minimally obtrusive survey produced usable data, a milestone product in the community of Sarasota County.

Increasing the proportion of youth meeting recommended physical activity guidelines is an essential outcome in the fight against obesity. Active youth are more likely to be active adults (42). Regular physical activity for youth contributes to building and maintaining healthy bones and joints, controlling weight, building lean muscle, reducing fat, and preventing or delaying the development of high blood pressure (43). Therefore, a community strategy for monitoring physical activity among tweens that capitalizes on the leadership and cooperation of multiple organizations may inspire methods for motivating youth to try new games and sports that they can sustain through the adolescent years and beyond.

## Figures and Tables

**Table 1:** Mean Number of Days Per Week Engaged in Vigorous-Intensity Physical Activity (VPA), by Sex and Grade Level, Students (N = 1,407) in Grades 5 Through 7, Sarasota County, Florida, May 2005

**Characteristic**	No. of Students (%)	Mean No. of Days Engaged in VPA (95% CI)
**Sex**		
Male^a^	687 (48.8)	5.22 (5.07-5.37)
Female	720 (51.2)	4.35 (4.20-4.50)
**Grade level**		
5th	315 (22.4)	4.86 (4.64-5.08)
6th^b^	578 (41.1)	4.93 (4.76-5.10)
7th	514 (36.5)	4.54 (4.35-4.73)

Abbreviation: CI, confidence interval.

a Boys reported significantly more days engaged in VPA than did girls (*P* < .001).

b Sixth-grade students reported significantly more days engaged in VPA than did 7th-grade students (*P* = .006).

**Table 2:** Number of Days Per Week Engaged in Vigorous-Intensity Physical Activity (VPA), Students (N = 1,407) in Grades 5 Through 7, Sarasota County, Florida, May 2005

**No. of Days Per Week Engaged in VPA**	No. of Students (%)
7	445 (31.6)
6	174 (12.4)
5	214 (15.2)
4	191 (13.6)
3	145 (10.3)
2	107 (7.6)
1	75 (5.3)
0	56 (4.0)

**Table 3:** Mean Number of Days Per Week Engaged in Vigorous-Intensity Physical Activity (VPA), by Selected Survey Items, Students (N = 1,407) in Grades 5 Through 7, Sarasota County, Florida, May 2005

**Survey Item**	No. of Students (%)	Mean No. of Days Engaged in VPA (95% CI)
**Have friends who play a game or sport every day**		
Most or all^a^	648 (46.1)	5.53 (5.39-5.67)
Some	608 (43.2)	4.31 (4.15-4.47)
None or just a few	151 (10.7)	3.39 (3.06-3.72)
**Tried a new game or sport in the last 2 months**		
Yes^b^	825 (58.6)	5.14 (5.01-5.27)
No	582 (41.4)	4.25 (4.07-4.43)
**Seen, read, or heard of VERB messages or ads**		
Yes^c^	1,250 (88.8)	4.82 (4.70-4.94)
No	157 (11.2)	4.37 (4.03-4.71)
**Seen, read, or heard of VERB Summer Scorecard messages or ads**		
Yes^d^	385 (27.4)	4.99 (4.79-5.19)
No	1,022 (72.6)	4.69 (4.56-4.82)
**Total**	1,407 (100.0)	4.77 (4.66-4.88)

Abbreviation: CI, confidence interval.

a Having friends who reported playing a game or sport every day was significantly correlated (*r* = .369, *P* < .001) with number of days engaged in VPA.

b Youth who tried a new game or sport in past 2 months reported significantly more days engaged in VPA than youth who did not (*P* < .001).

c Students who reported VERB awareness engaged in VPA a significantly higher mean number of days per week than did students who were unfamiliar with VERB (*P =* .01).

d Students who reported familiarity with VERB Summer Scorecard engaged in VPA a significantly higher mean number of days per week than did students who were unfamiliar with this program (*P =* .02).
